# Association of serum chemerin with calcium, alkaline phosphatase and bone mineral density in postmenopausal females

**DOI:** 10.12669/pjms.37.2.3907

**Published:** 2021

**Authors:** Saba Tariq, Sundus Tariq, Muhammad Shahzad

**Affiliations:** 1 Saba Tariq, MBBS, M.Phil. Associate Professor, Pharmacology, University Medical & Dental College, Faisalabad-38000, Pakistan. PhD Research Scholar, Pharmacology, University of Health Sciences, Lahore, Pakistan.; 2 Sundus Tariq, MBBS, M.Phil. Associate Professor, Physiology, University Medical & Dental College, Faisalabad-38000, Pakistan. PhD Research Scholar, Physiology, University of Health Sciences, Lahore, Pakistan; 3 Muhammad Shahzad, M.Phil, PhD. Professor/Head of Department, Pharmacology, University of Health Sciences, Lahore, Pakistan

**Keywords:** Chemerin, Alkaline Phosphatase, Calcium, Postmenopausal females

## Abstract

**Objectives:**

To investigate the association of serum chemerin with calcium, alkaline phosphatase and bone mineral density in postmenopausal non-osteoporotic and osteoporotic females.

**Methods:**

This cross-section analysis was carried out at the orthopedic department of Madina Teaching Hospital, Faisalabad, Pakistan, in the year 2017-2019. Postmenopausal females were divided into two groups according to their bone mineral density (BMD). All osteoporotic females had a T-score of -2.5 or less. Data were analyzed on SPSS-24.

**Results:**

A total of 140 women were included in our study (80 osteoporotic and 60 non-osteoporotic). Non significant difference in age and BMI was observed between osteoporotic and non-osteoporotic subjects (p=0.152) and (p=0.291) respectively. There was a significant difference found in total BMD, serum chemerin levels between osteoporotic and non-osteoporotic subjects p<0.001 in both parameters. No significant correlation of serum chemerin was found with serum calcium, serum alkaline phosphatase and BMD in postmenopausal osteoporotic females (p=0.907), (p=0.318) (p=0.664) respectively. A significant negative correlation was found between serum alkaline phosphatase levels and total BMD in postmenopausal osteoporotic females (p=- 0.039). Linear regression analysis of serum alkaline phosphatase levels with total BMD showed no association between BMD and serum alkaline phosphatase levels (p=0.869).

**Conclusion:**

There is no association of serum chemerin with calcium, ALP and bone mineral density in non-osteoporotic and osteoporotic postmenopausal females.

## INTRODUCTION

Chemerin is an adipokine that regulates the formation of fat cells (adipocytes) from stem cells. It is also involved in the maturation of adipocytes primarily through chemokine activation like receptor-1 (CMKLR1). In individuals with obesity, Type-2 diabetes, and osteoporosis, elevated levels of chemerin were identified.^1^

It is believed that Chemerin is not only involved in controlling the growth of adipocytes and the metabolism of glucose, but it also modifies metabolic functions in mature adipocytes.^2^ Besides, experimental evidence suggested that chemerin and its CMKLR1 receptor can play an important role in osteoblastogenesis, bone mineralization, and osteoclastogenesis inhibition.^3^ Few studies have explored the associations in humans between circulating levels of chemerin and bone mineral density (BMD) or osteoporosis. 4-6 In one study, inverse relationships between serum chemerin and lumbar BMD levels have been reported in obese postmenopausal women.^5^ However, there is contradictory evidence since serum levels of chemerin were higher^7^ or lower^4^ relative to healthy control subjects in osteoporosis patients.

Calcium determines the skeletal system’s preservation and is one of the key minerals involved in bone homeostasis. In all ethnic groups, increased calcium intake is associated with an increased bone mineral accretion rate to a threshold level. Similarly, it plays an important role in preventing bone loss and osteoporotic fractures in later life.^8^

Serum alkaline phosphatase (ALP) is the biomarker of bone formation that is most widely used in routine screening tests. ALP is a universal enzyme that plays an important role in forming osteoids and bone mineralization.^9^ Previous studies indicated an inverse relationship between adipokines and alkaline phosphatase.^10^,^11^ In a recently conducted research, a positive association was found between chemerin and liver enzymes.^12^

Further clarification on the cellular, molecular, and genetic basis of chemerin and its receptor is required to understand its role. It is important to establish the role of chemerin in the pathophysiology of disease so that it could be used as a diagnostic and therapeutic marker for several diseases.^13^ However, we could not find any study after literature search of last ten years in which serum chemerin and calcium association was seen in postmenopausal females; therefore, the present study was designed to investigate the association of serum chemerin with calcium, alkaline phosphatase and bone mineral density in postmenopausal non-osteoporotic and osteoporotic females.

## METHODS

In this cross-section analysis,140 postmenopausal females were selected. The sample size was calculated with 90% power of the study, 95% confidence level, using the formula: n=2S^2^ (Zα + Zβ)^2^/d^2^. These post-menopausal females were divided into two groups. Group-A consisted of 60 postmenopausal non-osteoporotic females (T-score ≥-1.0). Group-B consisted of 80 postmenopausal osteoporotic females. More cases were recruited to increase the precision of the study. All osteoporotic females had a T-score of -2.5 or less.

The postmenopausal females were selected from the orthopedic department of Madina Teaching Hospital in the year 2017-2018. The required sample size was taken after taking into consideration the stringent inclusion and exclusion criteria in mind. Females between 50 and 70 years of age, with menopause for more than one year and required T-scores, were included. Females with early menopause, liver or kidney failure, parathyroid disorders, and drugs that can interfere with bone metabolism were excluded from the study. Ethical approval for the research was received on 20-02-2017 from the University of Health Sciences, Lahore, Pakistan’s Ethical Review Committee.

After obtaining written informed consent, information was collected from all participants in a specially designed proforma. Anthropometric measurements, including height and weight, were calculated using standardized equipment. The body mass index (BMI) was calculated using standard formula. BMD was evaluated through DEXA scan, and serum parameters, including chemerin, alkaline phosphatase, and calcium, were performed at the postgraduate laboratory of the University Medical and Dental College, The University of Faisalabad with the help of commercially available kits. Serum chemerin levels were quantified by human chemerin enzyme-linked immunosorbent assay (ELISA), formulated by ELAB Sciences, USA whereas by the colorimetric method, serum calcium and alkaline phosphatase were measured using Roche diagnostics, Cobas 6000, COBI-CD, developed by Hitachi High Technologies Corporation, Tokyo, Japan.

### Statistical analysis

IBM-SPSS version 24 (Statistics Package for Social Sciences) was used for data processing. Shapiro-Wilk statistics have been used to verify the normality of the results. Mean±SD and median with IQR were provided for both normal and non-normally distributed quantitative variables. The Mann-Whitney U test was used to compare quantitative variables between the two groups. Correlation between serum chemerin, calcium, alkaline phosphatase, and BMD was seen using spearman’s correlation. A simple linear regression analysis was conducted to predict bone mineral density (T score). P-value ≤ 0.05 was taken as statistically significant.

## RESULTS

A total of 140 women were included in our study (80 osteoporotic and 60 non-osteoporotic). Non significant difference in age and BMI was observed between osteoporotic and non-osteoporotic subjects with p=0.152 and p=0.291 respectively. There was a significant difference found in total BMD, serum chemerin levels between osteoporotic and non-osteoporotic subjects p<0.001 in both parameters. No significant difference was observed in serum calcium and alkaline phosphatase levels between the groups (Table-I).

**Table-I:** General, anthropometric and biochemical parameters of study groups

*Parameters*	*Non-osteoporotic (n=60)*	*Osteoporotic (n=80)*	*p-value*
Age (years)	57 (50-68)	57 (50-69)	0.152
Menopausal age (years)	50 (47-58)	50 (45-59)	0.314
Height (m)	1.55 (1.37-1.65)	1.52 (1.37-1.63)	0.458
Weight (kg)	65.50 (35-122)	68 (39-111)	0.245
BMI (kg/m^2^)	27.82 (15.41-46.17)	28 (17.14-46.24)	0.291
Total BMD (g/cm^2^)	0.25 (-1.00 to 2.28)	-2.14 (-3.58 to -0.52)	<0.001*
Serum Chemerin (ng/mL)	0.15 (0.05-1.61)	0.23 (0.06-9.45)	<0.001*
Serum Calcium (mg/dL)	9.5 (7.8-10.6)	9.6 (8.7-12)	0.727
Serum Alkaline Phosphatase U/L	97 (53-159)	95 (57-169)	0.833

Values are given as median (IQR),

* p-value ≤ 0.05 is considered statistically significant.

**Fig.1:**
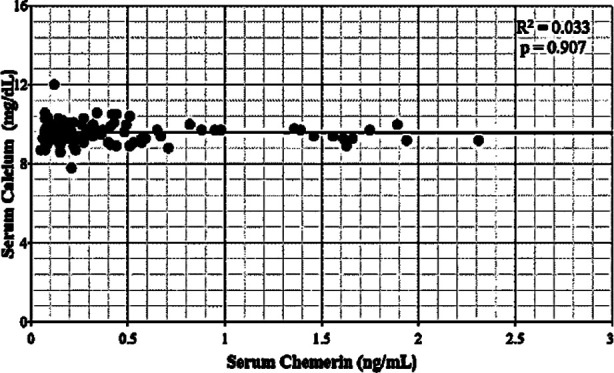
Scatter plot showing no correlation between serum chemerin and serum calcium in postmenopausal osteoporotic females using spearman`s rho correlation coefficient

No significant correlation was found between serum chemerin and calcium levels in postmenopausal normal and osteoporotic women (p=0.459) and (p=0.907) respectively. (Fig.1). Similarly, no correlation of serum chemerin was found with serum alkaline phosphatase (p=0.909 vs p=0.318) and total BMD (p=0.782 vs p=0.664) in postmenopausal normal and osteoporotic females respectively.

**Fig.2:**
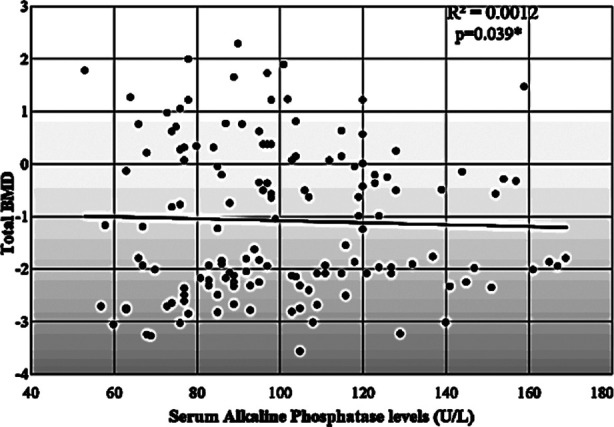
Scatter plot showing a Signiant negative correlation between serum alkaline phosphatase and Total BMD in postmenopausal females using spearman`s rho correlation coefficient

A significant negative correlation was found between serum alkaline phosphatase levels and total BMD in postmenopausal women (p=-0.039) (Fig.2).

Linear regression analysis of serum alkaline phosphatase levels with total BMD showed no association between BMD and serum alkaline phosphatase levels (p=0.869) (Table-II).

**Table-II:** Linear regression analysis of serum Alkaline Phosphatase levels with total Bone Mineral Density.

*Dependent variable*	*Model*	*Predictors*	*β coefficient*	*Std. Error*	*R*	*p-value*
Total BMD	1	Constant	-1.164	0.482	-	0.017
		Alkaline Phosphatase	0.001	0.005	0.014	0.869

p-value ≤ 0.05 is considered statistically significant

## DISCUSSION

Osteoporosis is a skeletal condition associated with a major health and economic burden and is becoming a growing global health issue. A number of human studies have previously indicated that adipokines, for example, leptin and resistin, might have an association with bone mineral density.^14^-^16^ In Pakistan the prevelance of osteoporosis is increasing at an alarming rate due to multiple factors. Therefore, it is important to find new diagnostic and therapeutic targets to treat this disease effectively.^17^

In this study, we found that serum chemerin was increased in the osteoporotic group compared to normal control, indicating that serum chemerin might be involved in osteoporosis’s pathogenesis. Similar to our results, another study reported an increased level of serum chemerin in patients with osteoporosis.^7^ In vitro studies suggested that chemerin shift differentiation of mesenchymal stem cells towards adipogenesis at the expense of osteoclastogenesis which shows a definitive harmful role of chemerin in bone homeostasis.^18^

However, contradictory data is available regarding chemerin effects on bone homeostasis as a recently conducted in vitro research concluded that treatment with chemerin prevents bone loss by inhibiting the formation and activity of osteoclast in breast cancer cells invading the bone tissue.^19^ Similarly, another study showed that chemerin can be used therapeutically to target osteoporosis due to its bone formation activity and inhibition of bone resorption.^20^

Chemerin is also one of the contributing factors in the pathogenesis of metabolic syndrome, and it had already been reported that the incidence of osteoporosis is increased in metabolic syndrome.^7^ Another study reported an increased level of chemerin in elderly females. However, they saw the levels of chemerin specifically in females with iron deficiency anemia.^21^

Given the difference in in vitro results regarding chemerin and bone new observational studies in human are important to explore the definitive role of chemerin in bone homeostasis. Our study bridges this gap and provides an insight in bone homeostasis, which may be context-specific, and elaborates role of chemerin in relation to bone metabolism.

According to the present study results, we could not find any association of serum chemerin with calcium, bone mineral density and alkaline phosphatase in postmenopausal females. To our knowledge, it is the first documented study at the level of Pakistan that has investigated whether serum chemerin had any association with calcium, bone mineral density and alkaline phosphatase in patients with osteoporosis. Although one of the studies suggested chemerin increases intracellular calcium concentration through activation of its receptors;^2^ however, there is a scarcity of data available regarding serum chemerin and calcium concentration. Similarly, few studies in the past had explored an inverse relationship between serum chemerin and alkaline phosphatase,^10^,^11^ but we could not find such an association in postmenopausal females. One interesting finding of our research was a significant negative association of serum alkaline phosphatase with bone mineral density. Still, linear regression analysis of serum alkaline phosphatase levels with total BMD showed no association between BMD and serum alkaline phosphatase levels. Contradictory data is available regarding serum alkaline phosphatase association with a bone mineral density as few studies reported inverse relationship,^22^,^23^ and other indicated no relationship.^24^ A recently conducted study found that serum ALP was the predictor of BMD in osteopenic females but not in osteoporotic females.^23^

### Limitation of the study

Small sample size was one limitation of this study. However, as no previous data is available so further studies with large sample size involving both genders can be conducted.

## CONCLUSION

There is no association of serum chemerin with calcium, bone mineral density and ALP. Serum chemerin was more in osteoporotic group as compare to non-osteoporotic group. Further studies on different populations with increase sample size are required to derive a definitive conclusion and to bridge this gap.

### Authors’ Contribution:

**Saba Tariq:** Designed the study, data entry, manuscript writing.

**Sundus Tariq:** Statistical analysis and interpretation of data.

**Muhammad Shahzad:** Supervised research. Review and final approval of the manuscript.

All the authors are responsible and accountable for the accuracy or integrity of the work.
